# Evaluation of the association between predictive factors and the development of immune‐related adverse events and prognostic factors for chemoimmunotherapy in patients with non‐small cell lung cancer: A multicenter retrospective study

**DOI:** 10.1002/cam4.70080

**Published:** 2024-08-03

**Authors:** Ryota Ozawa, Kei Sonehara, Tsutomu Hachiya, Shuhei Nozawa, Toshihiko Agatsuma, Hiroshi Yamamoto, Akane Kato, Akemi Matsuo, Taro Hirabayashi, Taisuke Araki, Masamichi Komatsu, Kazunari Tateishi, Masayuki Hanaoka

**Affiliations:** ^1^ Department of Respiratory Medicine Nagano Red Cross Hospital Nagano Japan; ^2^ Department of Internal Medicine Shinshu University School of Medicine Matsumoto Japan; ^3^ Department of Respiratory Medicine Suwa Red Cross Hospital Suwa Japan; ^4^ Department of Respiratory Medicine Nagano Municipal Hospital Nagano Japan; ^5^ Department of Respiratory Medicine National Hospital Organization Shinshu Ueda Medical Center Ueda Japan; ^6^ Department of Respiratory Medicine Iida Municipal Hospital Iida Japan; ^7^ Department of Respiratory Medicine Ina Central Hospital Ina Japan; ^8^ Department of Respiratory Medicine, Minaminagano Medical Center Shinonoi General Hospital Nagano Japan

**Keywords:** albumin, immune‐related adverse event, non‐small cell lung cancer, prognostic factor

## Abstract

**Introduction:**

Chemoimmunotherapy is widely used as the first‐line management of advanced non‐small cell lung cancer (NSCLC) in clinical settings. However, predictive factors associated with the development of immune‐related adverse events (irAEs) and prognostic factors for NSCLC patients undergoing chemoimmunotherapy remains largely unexplored. Therefore, in this study, we aimed to evaluate predictive factors for irAE development and prognostic factors associated with chemoimmunotherapy in NSCLC patients.

**Methods:**

This study enrolled 199 patients with advanced and recurrent NSCLC who underwent chemoimmunotherapy across eight institutions in Nagano prefecture from December 2018 to January 2023. We examined predictive factors associated with irAE development and prognostic factors associated with overall survival (OS).

**Results:**

Among the patients, 106 experienced irAEs, while 93 patients did not. A total of 44 (22.1%) patients developed multiple irAEs. High serum albumin levels (Alb >3.5 g/dL) emerged as an independent predictive factor associated with irAE development in logistic regression analysis (odds ratio; 2.35, 95% confidence interval 1.27–4.34, *p* = 0.007). Furthermore, the development of multiple irAEs (*p* = 0.016), lower lactate dehydrogenase level (<223 U/L, *p* = 0.002), and decreased neutrophil‐to‐lymphocyte ratio (<3, *p* = 0.049) were identified as independent favorable prognostic factors associated with OS in multivariate Cox hazard analyses.

**Conclusion:**

The study results suggest that high serum Alb is a predictive factor for irAE development and that the presence of multiple irAEs is a favorable prognostic indicator for NSCLC patients undergoing chemoimmunotherapy.

## 1 INTRODUCTION

The development and approval of immune checkpoint inhibitors (ICIs) for advanced non‐small cell lung cancer (NSCLC) have significantly improved patient prognosis. Recently, chemoimmunotherapy (comprising anti‐programmed death 1 [PD‐1]/programmed death ligand 1 [PD‐L1] antibody plus cytotoxic anticancer agents, and anti‐cytotoxic‐T‐lymphocyte‐associated protein 4 [CTLA‐4] antibody plus anti‐PD‐1/PD‐L1 antibody plus cytotoxic anticancer agents) has emerged as a first‐line treatment for advanced NSCLC, demonstrating efficacy in multiple phase III trials.^1^, ^2^, ^3^, ^4^, ^5^, ^6^, ^7^ ICIs enhance immune system function, leading to clinical benefits, but immune checkpoint blockade can induce inflammatory side effects known as immune‐related adverse events (irAEs). These irAEs can affect various organs or tissues, with the skin, colon, endocrine organs, liver, and lungs being commonly affected. Although less frequent, irAEs in other organs, such as neurological disorders and myocarditis, can be severe, even fatal.^8^ The incidence of irAEs varies depending on the type of ICI used and whether it is combined with cytotoxic anticancer agents. In trials such as CheckMate 017^9^ and CheckMate 057,^10^ irAEs occurred in 58%–69% of patients receiving nivolumab, with 7%–10% being grade 3 or 4. Combination therapy with nivolumab and ipilimumab has been associated with incidences of irAE, with CheckMate 227 reporting any irAE in 76.7% of patients and 32.8% being grade 3 or 4.^11^ In trials such as IMpower130, IMpower132, and IMpower150, chemoimmunotherapy with atezolizumab resulted in irAE incidence of ranging 45%–60%.^12^ Studies have suggested a correlation between irAEs and improved survival outcomes in various advanced malignant neoplasms, including NSCLC.^12^, ^13^, ^14^ Moreover, Shimozaki et al. demonstrated that the overall survival (OS) of patients with multiple irAEs was significantly longer than that of patients with a single irAE.^14^ Appropriate management of irAEs is crucial to optimize treatment outcomes, necessitating the identification of patients susceptible to irAEs. Several studies have investigated predictive factors for irAEs focusing on immunotherapy, and several risk factors have been reported, such as a low neutrophil‐to‐lymphocyte ratio (NLR), high body mass index (BMI), high serum albumin (Alb) level, and low Eastern Cooperative Oncology Group (ECOG) performance status (PS).^15^, ^16^, ^17^, ^18^, ^19^ However, limited data exist regarding chemoimmunotherapy. Fujimoto et al. suggested squamous cell carcinoma, anti‐PD‐1 plus anti‐CTLA‐4 regimens, and NLR <3 as potential predictive factors for irAEs during chemoimmunotherapy induction with platinum agents in patients with NSCLC.^20^ Nevertheless, further clinical evidence is warranted. Thus, this study aimed to evaluate predictive factors for irAE development and prognostic factors associated with chemoimmunotherapy in NSCLC patients.

## 2 MATERIALS AND METHODS

### 2.1 Patient selection

**FIGURE 1:**
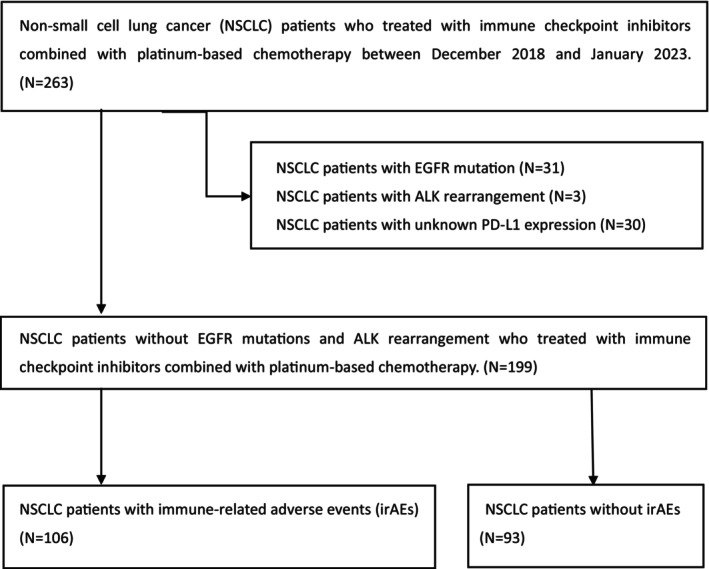
Patient selection consort diagram.

The CONSORT diagram showing patient selection is presented in Figure 1. This multicenter retrospective study was conducted at eight institutions in Nagano Prefecture. A total of 263 NSCLC patients who received immune checkpoint inhibitors combined with platinum‐based chemotherapy between December 2018 and January 2023 were initially included in the study database. Patients with NSCLC with epidermal growth factor receptor oncogenes, anaplastic lymphoma kinase rearrangement, and unknown PD‐L1 expression were excluded from the analysis. Ultimately, 199 NSCLC patients were enrolled in the study, comprising 106 (53.3%) patients who experienced irAEs and 93 (46.7%) who did not. Data collection was concluded on April 30, 2023. All data were extracted from the electronic medical records of each participating institution.

Data collection methods and analyses were conducted in accordance with the principles outlined in the Declaration of Helsinki. The retrospective study protocol was reviewed and approved by the Research Ethics Committee of the Shinshu University School of Medicine (approval number: 5494). An opt‐out approach was adopted, wherein the patients could decline participation by completing a form available on our institute's official website. Hence, the requirement for written informed consent was waived.

### 2.2 Data collection

All patient data were retrospectively collected and assessed at the initiation of first‐line treatment. The median follow‐up time was 12.4 months. The median follow‐up time in patients with irAEs and those without irAEs was 14.1 and 11.7 months, respectively. The best objective response to chemoimmunotherapy was assessed using the Response Evaluation Criteria in Solid Tumors, version 1.1.^21^ The OS was calculated from the date of chemoimmunotherapy initiation to the date of death or last follow‐up visit. The objective response rate was defined as the sum of the complete response rate and partial response rate. Treatment efficacy assessments were conducted at the discretion of the attending physician.

### 2.3 Statistical analysis

OS analyses were conducted using the Kaplan–Meier method with log‐rank tests. Univariate and multivariate analyses were performed using logistic regression analysis to identify predictive factors for irAE development and Cox regression modeling to determine prognostic factors associated with OS. Variables deemed significant (*p* < 0.05) in the univariate analysis and clinically significant variables were included in the multivariate analysis. Statistical analysis was conducted using IBM SPSS Statistics, version 26.

## 3 RESULTS

### 3.1 Patient characteristics

Patient characteristics are summarized in Table 1. Among the cohort, 159 (79.9%) and 40 (20.1%) patients were <75 years old and ≥75 years old, respectively. Male patients constituted 162 (81.4%) and female patients were 37 (18.6%). PS distribution was as follows: 178 (89.4%) patients had a PS of 0–1, 21 (10.6%) patients had a PS of 2–3. Disease staging was as follows: 19 (9.5%) patients were at stage III, and 144 (72.4%) were at stage IV, and 36 (18.1%) had postoperative recurrence. Regarding chemoimmunotherapy regimens, 156 (78.4%) received pembrolizumab combination therapy, 24 (12.1%) received atezolizumab combination therapy, and 19 (9.5%) received nivolumab plus ipilimumab combination therapy. Furthermore, 189 (95.0%) patients received chemoimmunotherapy as first‐line treatment, and 10 (5.0%) received it as a second or later treatment.

**TABLE 1:** Patient characteristics.

Category	All patients, *N* (%)	Patients with irAEs, *N* (%)	Patients without irAEs, *N* (%)
Patients (*N*)	199	106	93
Age (years), <75/≥75	159 (79.9)/40 (20.1)	81 (76.4)/25 (23.6)	78 (83.9)/15 (16.1)
Sex, male/female	162 (81.4)/37 (18.6)	80 (75.5)/26 (24.5)	82 (88.2)/11 (11.8)
ECOG PS, 0–1/2–3	178 (89.4)/21 (10.6)	94 (88.7)/12 (11.3)	84 (90.3)/9 (9.7)
Smoking history, no/yes	26 (13.1)/173 (86.9)	16 (15.1)/90 (84.9)	10 (10.8)/83 (89.2)
BMI (kg/m^2^), <25/≥25	163 (81.9)/36 (18.1)	90 (84.9)/16 (15.1)	73 (78.5)/20 (21.5)
Histologic subtype, non‐Sq/sq	140 (70.4)/59 (29.6)	71 (67.0)/35 (33.0)	69 (74.2)/24 (25.8)
PD‐L1 TPS (%), <50/≥50	139 (69.8)/60 (30.2)	75 (70.8)/31 (29.2)	64 (68.8)/29 (31.2)
Staging (TNM 8th)			
III	19 (9.5)	10 (9.4)	9 (9.7)
IV	144 (72.4)	79 (74.5)	65 (69.9)
Recurrence	36 (18.1)	17 (16.0)	19 (20.4)
Treatment regimen			
Platinum + PEM + pembrolizumab	88 (44.2)	38 (35.8)	50 (53.8)
Platinum + nab‐PAC (PAC) + pembrolizumab	68 (34.2)	42 (39.6)	26 (28.0)
CBDCA + PAC+ BEV + atezolizumab	10 (5.0)	6 (5.7)	4 (4.3)
CBDCA + PEM + atezolizumab	7 (3.5)	3 (2.8)	4 (4.3)
CBDCA + nab‐PAC + atezolizumab	7 (3.5)	3 (2.8)	4 (4.3)
CBDCA + PEM + nivolumab + ipilimumab	13 (6.5)	10 (9.4)	3 (3.2)
CBDCA + PAC + nivolumab + Ipilimumab	6 (3.0)	4 (3.8)	2 (2.2)
Lines of treatment, first/second or later	189 (95.0)/10 (5.0)	102 (96.2)/4 (3.8)	87 (93.5)/6 (6.5)
Laboratory findings			
Alb (g/dL), ≥3.5/<3.5	123 (61.8)/76 (38.2)	75 (70.8)/31 (29.2)	48 (51.6)/45 (48.4)
CRP (mg/dL), ≥0.5/<0.5 (*N* = 194)	126 (64.9)/68 (35.1)	62 (60.2)/41 (39.8)	64 (70.3)/27 (29.7)
Hb (g/dL), ≥12/<12	149 (74.9)/50 (25.1)	81 (76.4)/25 (23.6)	68 (73.1)/25 (26.9)
LDH (U/L), ≥223/<223 (*N* = 195)	71 (36.4)/124 (63.6)	39 (37.1)/66 (62.9)	32 (35.6)/58 (64.4)
NLR, ≥3/<3 (*N* = 198)	127 (64.1)/71 (35.9)	69 (65.1)/37 (34.9)	58 (63.0)/34 (37.0)

Abbreviations: BEV, bevacizumab; BMI, body mass index; ECOG, Eastern Cooperative Oncology Group; irAEs, immune‐related adverse events; NLR, neutrophil‐to‐lymphocyte ratio; PAC, paclitaxel; PD‐L1, programmed cell death ligand 1; PEM, pemetrexed; PS, performance status; Sq, squamous; TPS, tumor proportion score.

### 3.2 Types and severity of irAEs

The types and severity of irAEs are detailed in Table 2. Hepatopathy, interstitial lung disease, and skin rash were the most common irAEs, occurring in 18.6% of patients. The frequency of grade 3 or higher irAEs was in the following order: interstitial lung disease (5.5%), adrenal insufficiency (3.5%), and skin rash (2.0%). Interstitial lung disease (14.1%) accounted for the most frequent treatment discontinuations due to irAEs. Additionally, 44 (22.1%) patients developed multiple irAEs, with the skin (59.1%), liver (50.0%), and lungs (43.2%)being the most common sites (Table 3).

**TABLE 2:** Types and severity of immune‐related adverse events.

IrAE	Any grade *N* (%)	Grade 1, *N* (%)	Grade 2, *N* (%)	Grade 3, *N* (%)	Grade 4, *N* (%)	Discontinuation of treatment *N* (%)
Hepatopathy	37 (18.6)	27 (13.6)	7 (3.5)	3 (1.5)	0 (0.0)	1 (0.5)
Interstitial lung disease	37 (18.6)	10 (5.0)	16 (8.0)	11 (5.5)	0 (0.0)	28 (14.1)
Skin rash	37 (18.6)	12 (6.0)	21 (10.5)	4 (2.0)	0 (0.0)	3 (1.5)
Colitis	19 (9.5)	7 (3.5)	10 (5.0)	2 (1.0)	0 (0.0)	4 (2.0)
Thyroid dysfunction	19 (9.5)	9 (4.5)	10 (5.0)	0 (0.0)	0 (0.0)	1 (0.5)
Adrenal insufficiency	11 (5.5)	0 (0.0)	4 (2.0)	7 (3.5)	0 (0.0)	1 (0.5)
Infusion reaction	8 (4.0)	3 (1.5)	4 (2.0)	0 (0.0)	1 (0.5)	0 (0.0)
Polymyalgia rheumatica	2 (1.0)	0 (0.0)	2 (1.0)	0 (0.0)	0 (0.0)	0 (0.0)
Myositis	1 (0.5)	0 (0.0)	1 (0.5)	0 (0.0)	0 (0.0)	1 (0.5)
Rhabdomyolysis	1 (0.5)	0 (0.0)	0 (0.0)	1 (0.5)	0 (0.0)	0 (0.0)

Abbreviation: IrAE, immune‐related adverse event.

**TABLE 3:** Type of multiple immune‐related adverse events.

Category	*N* (%)
Patients, *N*	44
Skin + other	26 (59.1)
Liver + other	22 (50.0)
Lung + other	19 (43.2)
Thyroid gland + other	14 (31.8)
Colon + other	12 (27.3)
Adrenal gland + other	9 (20.5)
Number of developments of irAEs	
Two	30 (68.2)
Three	9 (20.5)
Four or more	5 (11.4)

Abbreviation: IrAE, immune‐related adverse event.

### 3.3 Predictive factors associated with development of irAEs

Table 4 summarizes the predictive factors associated with the development of irAEs. In the multivariate analysis, a high serum Alb level was an independent predictive factor associated with irAE development (odds ratio: 2.35, 95% confidence interval [CI]: 1.27–4.34, *p* = 0.007).

**TABLE 4:** Predictive factors associated with development of immune‐related adverse events.

Category	Univariate			Multivariate		
	OR	95% CI	*p*‐value	OR	95% CI	*p*‐value
Age (years)						
<75	Ref.					
≥75	1.61	0.79–3.27	0.193			
Sex						
Male	Ref.			Ref.		
Female	2.42	1.12–5.23	0.024	2.14	0.97–4.72	0.060
ECOG PS						
0–1	Ref.			Ref.		
2–3	1.19	0.48–2.97	0.707	1.64	0.62–4.32	0.320
Smoking history						
No	Ref.					
Yes	0.68	0.29–1.58	0.367			
BMI (kg/m^2^)						
<25	Ref.					
≥25	0.65	0.31–1.34	0.243			
Histologic subtype						
Non‐Sq	Ref.					
Sq	1.42	0.77–2.62	0.267			
PD‐L1 TPS (%)						
<50	Ref.			Ref.		
≥50	0.91	0.50–1.67	0.766	1.04	0.55–1.96	0.900
Type of ICI						
PD‐1 or PD‐L1	Ref.			Ref.		
PD‐1 + CTLA‐4	2.68	0.93–7.75	0.069	2.26	0.75–6.81	0.149
Lines of treatment						
First	Ref.					
Second or later	0.57	0.16–2.08	0.394			
Alb (g/dL)						
<3.5	Ref.			Ref.		
≥3.5	2.27	1.27–4.07	0.006	2.35	1.27–4.34	0.007
CRP (mg/dL)						
<0.5	Ref.					
≥0.5	0.64	0.35–1.16	0.141			
Hb (g/dL)						
<12	Ref.					
≥12	1.19	0.63–2.26	0.593			
LDH (U/L)						
<223	Ref.					
≥223	1.07	0.60–1.92	0.818			
NLR						
<3	Ref.					
≥ 3	1.09	0.61–1.96	0.764			

Abbreviations: BMI, body mass index; CI, confidence interval; CTLA‐4, cytotoxic T‐lymphocyte associated antigen‐4; ECOG, Eastern Cooperative Oncology Group; ICI, immune checkpoint inhibitor; irAEs, immune‐related adverse events; NLR, neutrophil‐to‐lymphocyte Ratio; OR, odds ratio; PD‐1, programmed death receptor‐1; PD‐L1, programmed cell death ligand 1; PS, performance status; Ref, reference; Sq, squamous; TPS, tumor proportion score.

### 3.4 Survival time

**FIGURE 2:**
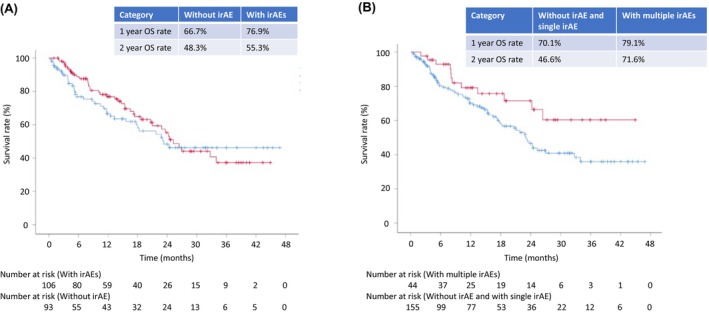
Kaplan–Meier curves. (A) The median overall survival in the without‐irAE group was not significantly different from that in the with‐irAE group (23.3 vs. 25.3 months, HR 0.87 [0.56–1.35], *p* = 0.539). (B) The median overall survival in the without‐irAE and single‐irAE groups was significantly shorter than that in the multiple‐irAE group (23.3 months vs. not reached, HR 0.54 (0.29–1.00), *p* = 0.049). irAE: immune‐related adverse events, HR: hazard ratio.

Kaplan–Meier curves of OS are presented in Figure 2. The median OS in NSCLC patients without irAEs did not significantly differ from those with irAEs (23.3 vs. 25.3 months; hazard ratio [HR]: 0.87, 95% CI: 0.56–1.35, *p* = 0.539). However, the median OS in NSCLC patients without irAEs and those with a single irAE was significantly shorter than in those with multiple irAEs (23.1 months vs. not reached; HR: 0.54, 95% CI: 0.29–1.00, *p* = 0.049). NSCLC patients with multiple irAEs exhibited a high 2‐year OS rate of 71.6%, compared with 46.6% for patients without irAEs or with a single irAE.

### 3.5 Prognostic factors associated with OS

Table 5 summarizes the prognostic factors associated with OS. The development of multiple irAEs (HR: 0.45, 95% CI: 0.24–0.86, *p* = 0.016), lower lactate dehydrogenase (LDH), and lower NLR were identified as independent favorable prognostic factors associated with OS. The irAE type was not a significant prognostic factor for OS (Table S1).

**TABLE 5:** Univariate and multivariate Cox hazard analyses of prognostic factors associated with overall survival.

Category	OS (months)	Univariate			Multivariate		
		HR	95% CI	*p*‐value	HR	95% CI	*p*‐value
Age, years							
<75 versus ≥75	24.2 versus 25.3	1.04	0.57–1.89	0.903			
Sex							
Male versus female	25.3 versus 22.9	1.15	0.67–2.00	0.612			
ECOG performance status							
0–1 versus 2–3	24.4 versus NR	0.93	0.45–1.94	0.850			
Smoking history							
No versus yes	23.6 versus 24.4	0.99	0.52–1.88	0.978			
BMI (kg/m^2^)							
<25 versus ≥25	23.6 versus 27.0	0.62	0.33–1.18	0.148			
Staging (TNM 8th)							
III and recurrence versus IV	NR versus 23.1	1.94	1.10–3.40	0.022	1.67	0.87–3.22	0.126
Histologic subtype							
Non‐sq versus sq	24.2 versus 27.0	0.94	0.57–1.55	0.799			
PD‐L1 TPS							
<50 versus ≥50	22.9 versus NR	0.68	0.41–1.11	0.121			
Type of ICI							
PD‐1 or PD‐L1 versus PD‐1 + CTLA‐4	24.4 versus NR	1.28	0.55–2.98	0.562			
Line of treatment							
First versus second or later	24.4 versus 21.0	1.07	0.39–2.92	0.902			
Development of irAEs							
Non + single versus multiple	23.1 versus NR	0.54	0.29–1.00	0.049	0.45	0.24–0.86	0.016
Alb (g/dL)							
<3.5 versus ≥3.5	17.7 versus 27.0	0.59	0.37–0.92	0.019	0.86	0.51–1.46	0.580
CRP (mg/dL)							
<0.5 versus ≥0.5	NR versus 22.8	1.72	1.03–2.87	0.038	1.39	0.78–2.47	0.259
Hb (g/dL)							
<12 versus ≥12	20.7 versus 27.0	0.70	0.42–1.16	0.165			
LDH (U/L)							
<223 versus ≥223	32.7 versus 16.5	1.81	1.15–2.83	0.010	2.12	1.32–3.41	0.002
NLR							
<3 versus ≥3	NR versus 22.9	1.90	1.16–3.12	0.011	1.76	1.00–3.08	0.049

Abbreviations: BMI, body mass index; CI, confidence interval; CTLA‐4, cytotoxic T‐lymphocyte associated antigen‐4; ECOG, Eastern Cooperative Oncology Group; HR, hazard ratio; ICI, immune checkpoint inhibitor; irAEs, immune‐related adverse events; NLR, neutrophil‐to‐lymphocyte ratio; NR, not reached; OS, overall survival; PD‐1, programmed death receptor‐1; PD‐L1, programmed cell death ligand 1; PS, performance status; Ref, reference; Sq, squamous; TPS, tumor proportion score.

## 4 DISCUSSION

This multicenter retrospective study revealed that high serum Alb level (≥3.5 g/dL) is a valuable predictive factor for the development of irAEs and that the development of multiple irAEs is a favorable prognostic factor for NSCLC patients undergoing chemoimmunotherapy.

The mechanism underlying irAE development is multifaceted, including cellular immune pathway due to activation of autoreactive T cells, humoral immune pathway by autoantibody production, and inflammatory pathways resulting from overproduction of inflammatory cytokines.^22^ Serum Alb serves not only as an important indicator reflecting overall ‘nutritional status but also plays a role in the activation of inflammatory cytokines. Elevated Alb level reduces prostaglandin E2 level, potentially facilitating the induction of inflammatory cytokines.^23^ Previous research has reported high Alb level as a possible predictive factor for the development of irAEs. In a retrospective study of 200 patients with carcinomas, including NSCLC, gastric cancer, and malignant melanoma, high Alb level (≥3.5 g/dL) was reported to be a predictive factor for the development of irAEs and a favorable prognostic factor for OS.^24^ In our study, the incidence of irAEs was higher in NSCLC patients with high Alb level, although high Alb level was not an independent favorable prognostic factor for OS. This discrepancy is probably due to the fact that not all irAEs correlate with survival outcomes. Our study found no significant difference in OS between patients with irAEs and those without irAE. Numerous studies have explored the correlation between individual irAEs and the prognosis of NSCLC patients undergoing immunotherapy. A previous meta‐analysis reported an association between thyroid dysfunction and favorable prognosis for NSCLC patients undergoing immunotherapy.^25^ Furthermore, a previous study reported dermatological adverse events as prognostic factors associated with survival outcomes in metastatic melanoma patients treated with anti‐PD‐1 antibody.^26^ However, the type of irAE that is a favorable prognostic factor may vary depending on cancer type and study population, necessitating further investigation.

This study also suggests that the development of multiple irAEs, along with LDH levels (<223 U/L) and lower NLR (<3), independently correlate with favorable prognostic factor associated with OS. These findings regarding LDH and NLR as prognostic factors for NSCLC patients treated with immunotherapy are consistent with previous reports.^27^, ^28^ Additionally, a retrospective study of 623 NSCLC patients treated with immunotherapy found that those experiencing multiple irAEs had significantly longer survival (progression‐free survival/OS) than those without irAEs.^29^ The development of multiple irAEs may indicate increased activation of autoreactive T cells compared with a single irAE and a higher complication rate of irAEs, which is associated with a better prognosis. The incidence of skin lesions (irAE) correlates with the prognosis of patients with malignant melanoma treated with immunotherapy.^30^ In our study, the highest rate of skin rash (59.1%) may have contributed to the favorable prognosis factor for OS. No significant difference was observed in OS based on irAE severity. The median OS did not significantly differ among NSCLC patients with Grade 1–2 irAEs (*p* = 0.464) or Grade 3–4 irAEs (*p* = 0.936) and NSCLC patients without irAEs (Figure S1). This contrasts with a previous that reported a significant difference in OS for NSCLC patients treated with immunotherapy between those experiencing mild and severe irAEs (34.3 vs. 17.3 months, *p* = 0.021).^31^ Regarding this study, it is possible that with a longer follow‐up period, OS differences could appear based on irAE severity. Thus, further follow‐up and evaluation are warranted.

This study has several limitations. First, this study was retrospective in nature and had a small sample size. Moreover, treatment regimen selection and treatment efficacy assessment were at the discretion of the attending physician. Second, the study did not examine the time until the development of individual irAEs. Third, the follow‐up period was insufficient to fully evaluate OS. Finally, the correlation between individual irAEs and survival time could not be examined.

In conclusion, high Alb level is a predictive factor for the development of irAEs, while the development of multiple irAEs is a favorable prognostic factor for NSCLC patients treated with chemoimmunotherapy. In the future, further verification of these results through prospective observational studies with longer follow‐up time is warranted.

## AUTHOR CONTRIBUTIONS

**Ryota Ozawa:** Conceptualization (lead); data curation (equal); formal analysis (equal); funding acquisition (equal); investigation (equal); methodology (equal); project administration (equal). **Kei Sonehara:** Conceptualization (lead); data curation (equal); formal analysis (lead); funding acquisition (equal); investigation (equal); methodology (equal); project administration (lead). **Tsutomu Hachiya:** Conceptualization (equal); data curation (equal); formal analysis (equal); funding acquisition (equal); investigation (equal); methodology (equal); project administration (equal). **Shuhei Nozawa:** Conceptualization (equal); data curation (equal); formal analysis (equal); funding acquisition (equal); investigation (equal); methodology (equal); project administration (equal). **Toshihiko Agatsuma:** Conceptualization (equal); data curation (equal); formal analysis (equal); funding acquisition (equal); investigation (equal); methodology (equal). **Hiroshi Yamamoto:** Conceptualization (equal); data curation (equal); formal analysis (equal); funding acquisition (equal); investigation (equal); methodology (equal); project administration (equal). **Akane Kato:** Conceptualization (equal); data curation (equal); formal analysis (equal); funding acquisition (equal); investigation (equal); methodology (equal); project administration (equal). **Akemi Matsuo:** Conceptualization (equal); data curation (equal); formal analysis (equal); funding acquisition (equal); investigation (equal); methodology (equal); project administration (equal). **Taro Hirabayashi:** Conceptualization (equal); data curation (equal); formal analysis (equal); funding acquisition (equal); investigation (equal); methodology (equal). **Taisuke Araki:** Conceptualization (equal); data curation (equal); formal analysis (equal); funding acquisition (equal); investigation (equal); methodology (equal); project administration (equal). **Masamichi Komatsu:** Conceptualization (equal); data curation (equal); formal analysis (equal); funding acquisition (equal); investigation (equal); methodology (equal); project administration (equal). **Kazunari Tateishi:** Conceptualization (equal); data curation (equal); formal analysis (equal); funding acquisition (equal); investigation (equal); methodology (equal). **Masayuki Hanaoka:** Conceptualization (equal); data curation (equal); formal analysis (equal); funding acquisition (equal); investigation (equal); methodology (equal); project administration (equal).

## FUNDING INFORMATION

N/A.

## CONFLICT OF INTEREST STATEMENT

The authors have no conflicts of interest to declare.

## ETHICS STATEMENT

All data collection and analyses were conducted in accordance with the principles of the Declaration of Helsinki. The retrospective study protocol was reviewed and approved by the Research Ethics Committee of the Shinshu University School of Medicine (approval number: 5494). An opt‐out method was used wherein the patients could decline participation by filling a form that was available on our institute's official website. Hence, the need for written informed consent was waived off.

## Supporting information


**Figure S1.** Kaplan–Meier curves. (A) The median overall survival in the without‐irAE group was not significantly different from that in the Grade 1–2 irAE group (23.3 vs. 25.3 months, HR 0.83 [0.51–1.36], *p* = 0.464). (B) The median overall survival in the without‐irAE group was not significantly different from that in the ≥Grade 3 irAE group (23.3 vs. 24.4 months, HR 0.98 [0.52–1.83], *p* = 0.936). irAE: immune‐related adverse events, HR: hazard ratio.

**Table S1.** Univariate Cox hazard analyses of prognostic predictors associated with overall survival.

## Data Availability

The data generated during and/or analyzed during the present study are available from the corresponding author upon reasonable request.
