# Supplementary material for: Evaluation of the association between predictive factors and the development of immune‐related adverse events and prognostic factors for chemoimmunotherapy in patients with non‐small cell lung cancer: A multicenter retrospective study
Source: Cancer Med. 2024 Aug 3;13(15):e70080. doi: 10.1002/cam4.70080 (PMC11297531; doi:10.1002/cam4.70080)
Supplement: Supplementary file 1 — Figure S1. Kaplan–Meier curves. (A) The median overall survival in the without‐irAE group was not significantly different from that in the Grade 1–2 irAE group (23.3 vs. 25.3 months, HR 0.83 [0.51–1.36], p = 0.464). (B) The median overall survival in the without‐irAE group was not significantly different from that in the ≥Grade 3 irAE group (23.3 vs. 24.4 months, HR 0.98 [0.52–1.83], p = 0.936). irAE: immune‐related adverse events, HR: hazard ratio. [file CAM4-13-e70080-s002.pptx]

## Slide 1
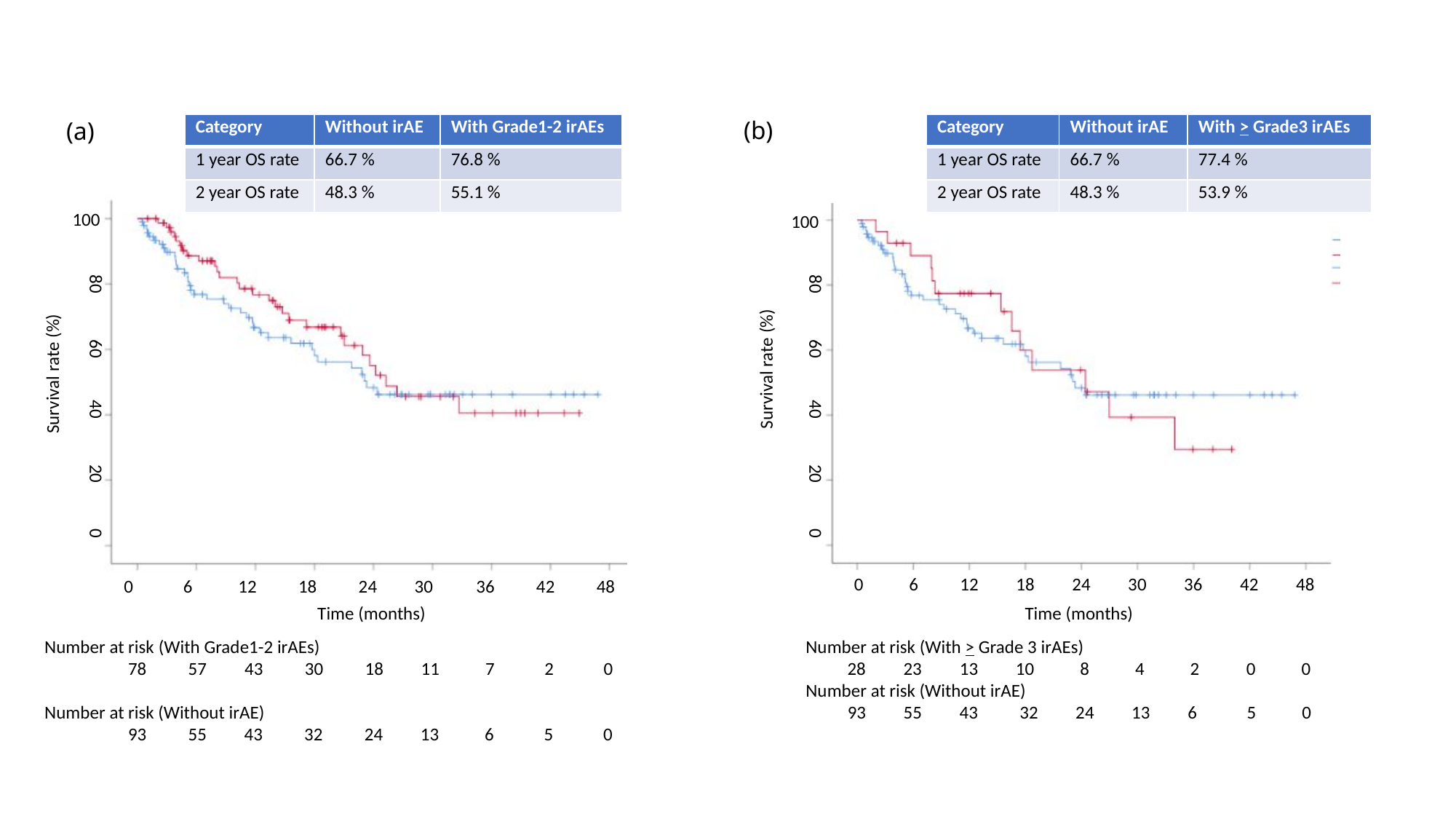

(b)
(a)
| Category | Without irAE | With Grade1-2 irAEs |
| --- | --- | --- |
| 1 year OS rate | 66.7 % | 76.8 % |
| 2 year OS rate | 48.3 % | 55.1 % |
| Category | Without irAE | With > Grade3 irAEs |
| --- | --- | --- |
| 1 year OS rate | 66.7 % | 77.4 % |
| 2 year OS rate | 48.3 % | 53.9 % |
100
100
 80 60 40 20 0
 80 60 40 20 0
Survival rate (%)
Survival rate (%)
 0 6 12 18 24 30 36 42 48
 0 6 12 18 24 30 36 42 48
Time (months)
Time (months)
Number at risk (With Grade1-2 irAEs)
 78 57 43 30 18 11 7 2 0
Number at risk (Without irAE)
 93 55 43 32 24 13 6 5 0
Number at risk (With > Grade 3 irAEs)
 28 23 13 10 8 4 2 0 0
Number at risk (Without irAE)
 93 55 43 32 24 13 6 5 0
