# Supplementary material for: Evaluation of the association between predictive factors and the development of immune‐related adverse events and prognostic factors for chemoimmunotherapy in patients with non‐small cell lung cancer: A multicenter retrospective study
Source: Cancer Med. 2024 Aug 3;13(15):e70080. doi: 10.1002/cam4.70080 (PMC11297531; doi:10.1002/cam4.70080)
Supplement: Supplementary file 2 — Table S1. Univariate Cox hazard analyses of prognostic predictors associated with overall survival. [file CAM4-13-e70080-s001.docx]

Supplemental table1. Univariate Cox hazard analyses of prognostic predictors associated with overall survival

| Category  (Type of immune-related adverse event) | | OS  (months) | Univariate | | |
| --- | --- | --- | --- | --- | --- |
|  |  |  | HR | 95% CI | p-value |
| Hepatopathy | No | 24.4 | Ref | | |
|  | Yes | 22.9 | 0.92 | 0.51-1.67 | 0.786 |
| Interstitial lung disease | No | 24.2 | Ref | | |
|  | Yes | 32.7 | 0.94 | 0.54-1.66 | 0.836 |
| Skin rash | No | 24.4 | Ref | | |
|  | Yes | 24.2 | 1.08 | 0.63-1.85 | 0.775 |
| Colitis | No | 23.6 | Ref | | |
|  | Yes | NR | 0.54 | 0.22-1.34 | 0.182 |
| Thyroid dysfunction | No | 24.2 | Ref | | |
|  | Yes | NR | 0.59 | 0.24-1.46 | 0.251 |
| Adrenal insufficiency | No | 23.6 | Ref | | |
|  | Yes | NR | 0.33 | 0.08-1.33 | 0.119 |

Abbreviations: OS, overall survival; NR, not reached; HR, Hazard Ratio; CI, Confidence Interval
